# Supplementary material for: Empirical methods for controlling false positives and estimating confidence in ChIP-Seq peaks
Source: BMC Bioinformatics. 2008 Dec 5;9:523. doi: 10.1186/1471-2105-9-523 (PMC2628906; doi:10.1186/1471-2105-9-523)
Supplement: Additional File 1 — A variety of html documents from the USeq web site detailing the available applications, their best usage, output file type descriptions, command line menus, etc. [file 1471-2105-9-523-S1.zip › USeqUserGuides/JavaDoc.zip!/JavaDoc/trans/main/IntervalGFFPrinter.html]

IntervalGFFPrinter


|  |  |  |
| --- | --- | --- |
| **Overview** \| **Package** \| **Class** \| **Use** \| **Tree** \| **Deprecated** \| **Index** \| **Help** | |  |
| **PREV CLASS**   **NEXT CLASS** | **FRAMES**    **NO FRAMES**     **All Classes** |  |
| SUMMARY: NESTED \| FIELD \| CONSTR \| METHOD | DETAIL: FIELD \| CONSTR \| METHOD |  |


---


## trans.main Class IntervalGFFPrinter

```
java.lang.Object
  trans.main.IntervalGFFPrinter
```

---

``` public class IntervalGFFPrinter extends java.lang.Object ```

Prints intervals as a GFF3 File.

---

**Constructor Summary**

`IntervalGFFPrinter(java.lang.String[] args)`


| **Method Summary** | |
| --- | --- |
| `static void` | `gff(BindingPeak peak, Interval i, java.lang.String name, int peakNumber, java.lang.StringBuffer s)`             Appends a GFF3 line for a BindingPeak |
| `void` | `gff(Interval i, int rank, java.lang.String name, java.lang.StringBuffer s)`             Appends a GFF3 line for an Interval. |
| `void` | `gff(Interval i, java.lang.String name, java.lang.StringBuffer s)`             Appends a GFF3 line for a Window. |
| `static void` | `gff(Oligo o, Interval i, java.lang.String name, int oligoNumber, java.lang.StringBuffer s)`             Appends a GFF3 line for an Oligo. |
| `static void` | `gff(SubWindow sub, Interval i, java.lang.String name, java.lang.StringBuffer s)`             Appends a GFF3 line for a SubWindow. |
| `static void` | `main(java.lang.String[] args)` |
| `static void` | `printDocs()` |
| `void` | `printGFF()` |
| `void` | `processArgs(java.lang.String[] args)`             This method will process each argument and assign any new varibles |

**Methods inherited from class java.lang.Object**

`equals, getClass, hashCode, notify, notifyAll, toString, wait, wait, wait`

**Constructor Detail**

### IntervalGFFPrinter

```
public IntervalGFFPrinter(java.lang.String[] args)
```


**Method Detail**

### printGFF

```
public void printGFF()
```

---


### gff

```
public static void gff(Oligo o,
                       Interval i,
                       java.lang.String name,
                       int oligoNumber,
                       java.lang.StringBuffer s)
```

:   Appends a GFF3 line for an Oligo. Summary Score is a log2Ratio

---


### gff

```
public static void gff(BindingPeak peak,
                       Interval i,
                       java.lang.String name,
                       int peakNumber,
                       java.lang.StringBuffer s)
```

:   Appends a GFF3 line for a BindingPeak

---


### gff

```
public static void gff(SubWindow sub,
                       Interval i,
                       java.lang.String name,
                       java.lang.StringBuffer s)
```

:   Appends a GFF3 line for a SubWindow. Summary score is median ratio

---


### gff

```
public void gff(Interval i,
                int rank,
                java.lang.String name,
                java.lang.StringBuffer s)
```

:   Appends a GFF3 line for an Interval.

---


### gff

```
public void gff(Interval i,
                java.lang.String name,
                java.lang.StringBuffer s)
```

:   Appends a GFF3 line for a Window.

---


### printDocs

```
public static void printDocs()
```

---


### processArgs

```
public void processArgs(java.lang.String[] args)
```

:   This method will process each argument and assign any new varibles

---


### main

```
public static void main(java.lang.String[] args)
```


---


|  |  |  |
| --- | --- | --- |
| **Overview** \| **Package** \| **Class** \| **Use** \| **Tree** \| **Deprecated** \| **Index** \| **Help** | |  |
| **PREV CLASS**   **NEXT CLASS** | **FRAMES**    **NO FRAMES**     **All Classes** |  |
| SUMMARY: NESTED \| FIELD \| CONSTR \| METHOD | DETAIL: FIELD \| CONSTR \| METHOD |  |


---
